# Healthy volunteers' perceptions of risk in US Phase I clinical trials: A mixed-methods study

**DOI:** 10.1371/journal.pmed.1002698

**Published:** 2018-11-20

**Authors:** Jill A. Fisher, Lisa McManus, Marci D. Cottingham, Julianne M. Kalbaugh, Megan M. Wood, Torin Monahan, Rebecca L. Walker

**Affiliations:** 1 Department of Social Medicine and Center for Bioethics, University of North Carolina at Chapel Hill, Chapel Hill, North Carolina, United States of America; 2 Department of Sociology and Anthropology, North Carolina State University, Raleigh, North Carolina, United States of America; 3 Department of Sociology, University of Amsterdam, Amsterdam, the Netherlands; 4 Department of Communication, University of North Carolina at Chapel Hill, Chapel Hill, North Carolina, United States of America; University of Toronto, CANADA

## Abstract

**Background:**

There is limited research on healthy volunteers’ perceptions of the risks of Phase I clinical trials. In order to contribute empirically to long-standing ethical concerns about healthy volunteers’ involvement in drug development, it is crucial to assess how these participants understand trial risks. The objectives of this study were to investigate (1) participants’ views of the overall risks of Phase I trials, (2) their views of the risk of personally being harmed in a trial, and (3) how risk perceptions vary across participants’ clinical trial history and sociodemographic characteristics.

**Methods and findings:**

We qualitatively and quantitatively analyzed semi-structured interviews conducted with 178 healthy volunteers who had participated in a diverse range of Phase I trials in the United States. Participants had collective experience in a reported 1,948 Phase I trials (mean = 10.9; median = 5), and they were interviewed as part of a longitudinal study of healthy volunteers’ risk perceptions, their trial enrollment decisions, and their routine health behaviors. Participants’ qualitative responses were coded, analyzed, and subsequently quantified in order to assess correlations between their risk perceptions and demographics, such as their race/ethnicity, gender, age, educational attainment, employment status, and household income. We found that healthy volunteers often viewed the overall risks of Phase I trials differently than their own personal risk of harm. The majority of our participants thought that Phase I trials were medium, high, or extremely high risk (118 of 178), but most nonetheless felt that they were personally safe from harm (97 of 178). We also found that healthy volunteers in their first year of clinical trial participation, racial and ethnic minority participants, and Hispanic participants tended to view the overall trial risks as high (respectively, Jonckheere-Terpstra, −2.433, *p* = 0.015; Fisher exact test, *p* = 0.016; Fisher exact test, *p* = 0.008), but these groups did not differ in regard to their perceptions of personal risk of harm (respectively, chi-squared, 3.578, *p* = 0.059; chi-squared, 0.845, *p* = 0.358; chi-squared, 1.667, *p* = 0.197). The main limitation of our study comes from quantitatively aggregating data from in-depth interviews, which required the research team to interpret participants’ nonstandardized risk narratives.

**Conclusions:**

Our study demonstrates that healthy volunteers are generally aware of and reflective about Phase I trial risks. The discrepancy in healthy volunteers’ views of overall and personal risk sheds light on why healthy volunteers might continue to enroll in clinical trials, even when they view trials on the whole as risky.

## Abstract 2

Jill A. Fisher and colleagues use mixed-methods analysis of 178 interviews to discover how US Phase I trial participants assess their personal risk when they choose to volunteer.

## Author summary

**Why was this study done?:**

- The purpose of this study was to better understand how healthy volunteers perceive the risks of enrolling in Phase I clinical trials. These trials offer healthy volunteers no direct medical benefit for their participation, but participants are financially compensated.
- Ethical concerns regarding undue inducement—or that the offer of substantial amounts of money may distort healthy volunteers’ risk perceptions and encourage them to enroll—have long been associated with Phase I trials because participants are typically underemployed and disproportionately members of minority groups. Little empirical research has explored how individuals participating in Phase I trials describe the risks.

**What did the researchers do and find?:**

- We conducted interviews with 178 healthy volunteers who had experience participating in a variety of US Phase I trials. We asked questions about their perceptions of trial risks, how they decide to enroll in such trials, and their everyday health-related activities.
- We took a mixed-methods approach to analyzing participants’ interview data to find qualitative themes in how they described Phase I risks and quantitative differences in how groups of healthy volunteers differed from each other.
- Our main finding was that healthy volunteers often distinguished the overall risks of Phase I trials from their own personal risk of harm: many saw trials in general to be highly or moderately risky, while feeling they were unlikely to be harmed personally. We also found that healthy volunteers who were new to clinical trial participation and minority participants, particularly Hispanics, tended to view the overall risks of trials as higher compared with those who had participated in clinical trials for a longer time and non-Hispanic whites.

**What do these findings mean?:**

- This study sheds light on how Phase I trial risks are understood by diverse healthy volunteers with a range of trial experiences. Many healthy volunteers hold the incongruous view that Phase I trials overall carry significant risk but that they are nevertheless personally safe from harm. This provides some indication of undue inducement in Phase I trials, given that healthy volunteers, particularly those who are new to clinical trials or members of minority groups, engage in improbable narratives of personal exemption from risk of harm when justifying their participation in what they typically see as a risky activity.
- Phase I investigators should question the extent to which recruitment and informed consent processes can address the gap between how healthy volunteers think about overall trial risks and their potential to be personally harmed in studies.

## Introduction

Healthy volunteers are recruited using financial incentives to participate in Phase I clinical trials to evaluate the safety and tolerability of investigational drugs. These trials are generally considered quite safe [1], which justifies the enrollment of healthy participants who cannot personally benefit medically from their involvement. Meta-analyses of Phase I trial risks indicate that healthy volunteers are highly likely to experience short-term bodily changes, such as headaches or gastrointestinal problems, but serious or life-threatening problems are rare [2–5]. Yet, when tragedies occur in Phase I trials, news stories about deaths or permanent injury to healthy volunteers often circulate widely [6–9].

While their economic motivation to enroll in Phase I trials is well documented [10–14], limited empirical research explores healthy volunteers’ risk perceptions. Prior scholarship suggests that healthy volunteers, many of whom are serial participants, generally view trial risks as low, both in terms of absolute risk [14,15] and relative risk, when taking into account other risks in their lives, such as those associated with poverty or living in urban environments [16]. This is not to say that healthy volunteers participate indiscriminately in Phase I trials; some studies have found that their enrollment decisions are based on specific trials’ risks [17,18]. Yet, when asked whether they would join studies requiring lumbar punctures or biopsies and studies cautioning that death or life-threatening reactions could occur, many healthy volunteers still expressed willingness to enroll [19]. Some scholars have explained this seeming contradiction in terms of how the financial compensation can lead healthy volunteers to ignore risk information and make poor-quality decisions about enrollment [20–22]. This raises the ethical question about whether healthy volunteers are unduly influenced by the financial compensation and enroll in Phase I trials despite their concerns about risk and even against their better judgment [23–25]. Thus, the extant literature underscores the need to study the range of factors influencing participants’ decision-making; these factors certainly include compensation but also healthy volunteers’ own understanding of both the overall risks of Phase I trials and their personal risk of being harmed.

Prior research has also demonstrated a general bias to view one’s own risk of being harmed as lower than others’ risk [26]. In particular, studies on perceptions of HIV vaccination highlight the range of views that individuals can hold when it comes to assessing overall versus personal risk [27–29]. Risk communication research has predominately focused on cognitive, knowledge-based assessments (or cognitive representations as mental models) [30], but research in public health and the social sciences is increasingly turning to the social and emotional underpinnings of biomedical risk perceptions [31,32]. To date, these approaches have been primarily qualitative or quantitative, with few studies using a mixed-methods approach.

Our study investigates healthy volunteers’ multifaceted risk perceptions. The actual Phase I trials in which participants had enrolled varied in terms of the types of investigational drugs being tested, study procedures used, and trial designs. We examine how participants reflect on Phase I trials as a whole, including their perceptions of (1) the overall risks of Phase I trials and (2) the risk of personally being harmed in a trial. Furthermore, using a mixed-methods approach that translated qualitative findings into quantitative categories, we compare healthy volunteers’ risk perceptions based on their clinical trial history and sociodemographic characteristics, such as their race/ethnicity, gender, age, educational attainment, employment status, and household income.

## Materials and methods

### Study design

This article uses data collected as part of the HealthyVOICES Project, a longitudinal, mixed-methods study of healthy volunteers who had participated in at least one Phase I trial (see [33] for a more detailed account of the study design). This study is reported as per the Consolidated Criteria for Reporting Qualitative Research (COREQ) guidelines (**S1 Checklist**). We identified healthy volunteers by recruiting them while they were enrolled in a Phase I trial at one of seven US research clinics. Diverse Phase I trials were taking place at these clinics during our recruitment visits, with some clinics simultaneously conducting multiple trials. All the trials involved testing a pharmaceutical but varied in their therapeutic areas, including but not limited to investigational drugs for cholesterol, autoimmune diseases, and psychiatric illnesses. The trials also varied in purpose and design, such as first-in-human trials, bioequivalence trials, and drug–drug interaction trials. Although the clinics gave permission for our research team to enroll healthy volunteers on-site, they were not involved in the design of the study or analyses of the data. The study was reviewed and approved by the Biomedical Institutional Review Board at the University of North Carolina at Chapel Hill (#13–1256). All research participants provided written informed consent.

We enrolled participants between May and December 2013 using a convenience sampling approach based on which healthy volunteers were enrolled in Phase I trials during our scheduled clinic visits. That said, to increase the generalizability of our sample, we selected clinics in different parts of the country, enrolling roughly one third of our sample on the East Coast, the Midwest, and the West Coast, respectively. All healthy volunteers who spoke either English or Spanish were eligible for our study. No prior relationships existed between members of the research team and any of the prospective participants.

Our all-female recruitment team included the HealthyVOICES principal investigator (PI), two postdoctoral fellows, and two graduate students (both of whom enrolled English- and Spanish-speaking participants). The PI was accompanied by 1–2 team members per clinic visit. In each clinic, we approached prospective participants and gave them information about the longitudinal study. Compensation, offered in the form of a US$20 Visa gift card, was based on completion of an initial interview, and participants were informed about the opportunity to receive up to US$450 for completing four additional interviews over the course of their 3-year involvement in our study. Approximately 10% of the healthy volunteers invited to participate declined.

### Data collection

After consent was obtained, participants provided demographic information about their gender, race, ethnicity, date of birth, employment status, educational attainment, and household income. We also took a brief clinical trial history that included the total number of Phase I trials completed, year of enrolling in first Phase I trial, and estimated total earnings from Phase I trial participation. Participants then took part in a “baseline” semi-structured interview. We report on this single wave of interviews here. The interview guide elicited participants’ perceptions of Phase I risks and benefits, their decision-making about trial enrollment, information about their health and health behaviors, and stories about their prior experiences in Phase I trials, including their perceptions of the clinics, staff, and study compensation (see **S1 Appendix**). As is typical for semi-structured interviews [34], the interview guide included open-ended questions that were generally applicable to all participants, but the interviewer adapted questions or asked follow-ups based on participants’ responses. Regarding risk perceptions, participants were asked about their current trial’s specific risks and the overall risks of Phase I trials. Risk perceptions also emerged in discussions about their decision-making about trial enrollment, experiences of adverse effects, and typical health behaviors, which often included information about posttrial routines. Interview length varied per participant based on their clinical trial history and tendency for brevity or verbosity; the average interview was approximately 70 minutes, with a range of 21 to 164 minutes.

### Analysis

No prospective analysis plan was formulated for these data. Instead, as with many qualitative studies, our goal was to engage the data through an “abductive” process that facilitates ground-up analysis to identify emergent themes while also building upon prior research [35]. Interviews were audio recorded, transcribed in full, and verified and corrected for accuracy. De-identified Spanish transcripts were translated into English either by members of the research team or a professional translator. The finalized transcripts were then uploaded to Dedoose qualitative software and were coded by two members of the research team to ensure rigor and thoroughness [36]. Relevant to this article, we developed codes to capture participants’ risk perceptions, including subcodes for their “overall” risk perception, specific-study risk perceptions, initial risk perception, and changes to their risk perceptions. The resulting data set included 2,018 risk-related excerpts from the 178 participants.

After coding, we quantitatively categorized each participant’s risk perception. Our method of “quantitizing” balanced “numerical precision with narrative complexity” and enabled comparison based on participants’ demographic characteristics ([37], p. 208). We exported from Dedoose all transcript excerpts coded as risk perceptions. We organized excerpts by participant to categorize each person holistically, taking into account each excerpt’s nuance while understanding them in the context of the participant’s whole interview. We began by categorizing participants’ perception of “overall risk,” or their sense of how risky Phase I trials are generally. Alongside multiple readings of the excerpts, we iteratively developed an ordinal scale of no risk, low risk, medium risk, high risk, extremely high risk, and missing data. In the process of categorizing participants, we noted that their perception of overall risk did not always correlate with their perception of whether they would personally be harmed from their trial participation. To account for these differing perceptions, we developed additional categorical variables focusing on participants’ statements about their personal risk of immediate harm during the clinical trial. The “personal harm” variable ultimately included safe from harm, vulnerable to harm, and missing data. We then used open-coding techniques to identify categories, or “rationales,” for how participants justified their views of their personal risk of harm [38]. These started as general descriptions, and we fine-tuned them into a standardized list of terms that we grouped under “safe from harm” or “vulnerable to harm,” respectively. Finally, we recommenced the process of holistically evaluating each participant for all three variables (i.e., overall risk, personal harm, and personal harm rationales). To do so, three team members categorized each participant independently, then met to review and adjudicate differences to come to a consensus on each participant’s categorization. Once completed, these variables were entered into the project database and linked to each participant’s record.

To analyze the overall risk data, we used Fisher exact tests for categorical demographic data and Jonckheere-Terpstra tests for ordinal demographic data. Jonckheere-Terpstra tests were conducted as part of the peer-review process, based on the recommendation of a test that offered more precision for ordinal demographic data. Pearson chi-squared tests were used to determine whether there were statistically significant correlations between the personal risk variables and participants’ demographic characteristics. Missing data were excluded from the analysis. We report on statistical differences at the significance level *p* < 0.05.

## Results

### Participant characteristics

Our sample of healthy volunteers included 178 individuals from a diverse range of racial/ethnic groups, ages, educational attainments, employment statuses, household incomes, and clinical trial experience (**Table 1**). As is typical of US Phase I trials [18,39], our sample was predominantly men (74%) and from racial and ethnic minority groups (68%). Fourteen participants were interviewed in Spanish. Additionally, most participants had prior Phase I trial experience, with only 21% enrolled in their first clinical trial. More than half (51.1%) had participated in at least five Phase I trials. Collectively, participants reported having enrolled in a total of 1,948 Phase I trials (mean = 10.9; median = 5).

**Table 1:** Demographics of study participants (*N* = 178).

Demographics	*n*	Percent
**Gender**		
Women	47	26.4%
Men	131	73.6%
**Clinical Trial Experience**		
1 study	38	21.3%
2–4 studies	49	27.5%
5–10 studies	45	25.3%
11–200 studies	46	25.8%
**Years Since First Trial**		
Less than 1	53	29.8%
1–5	71	39.9%
6–10	25	14.0%
11–15	18	10.1%
16–32	11	6.2%
**Race/Ethnicity**		
Non-Hispanic white	57	32.0%
Black/African American	72	40.4%
American Indian	2	1.1%
Asian	6	3.4%
Hawaiian/Pacific Islander	2	1.1%
More than one race	13	7.3%
Hispanic^1^	38	21.3%
**Age**		
18–21	6	3.4%
22–29	34	19.1%
30–39	58	32.6%
40–49	54	30.3%
50+	26	14.6%
**Household Income**^**2**^		
Less than US$10,000	30	16.9%
US$10,000 to US$24,999	52	29.2%
US$25,000 to US$49,999	71	39.9%
US$50,000 to US$74,999	13	7.3%
US$75,000 to US$99,999	7	3.9%
US$100,000 or more	4	2.2%
**Educational Attainment**		
Less than high school	12	6.7%
High school or GED	37	20.8%
Some college	52	29.2%
Trade/technical/vocational training	19	10.7%
Associate’s degree	21	11.8%
Bachelor’s degree	32	18.0%
Graduate degree	5	2.8%
**Employment Status**^**3**^		
Full-time/Business owner (self-employed)	45	25.3%
Part-time/Independent or Irregular Contractor	60	33.7%
Unemployed/Retired	73	41.0%

^1^The category Hispanic is composed of all racial groups. Our sample included Hispanic individuals identifying as white, black, more than one race, American Indian, and Native Hawaiian/Pacific Islander.

^2^Household income was not reported by one participant.

^3^These data are based on consolidated definitions of each employment category that we used to standardize participants’ self-reported data.

### Multifaceted nature of healthy volunteers’ risk perceptions

Building on prior research on risk perceptions [26,27], our analysis suggests that healthy volunteers often viewed the overall risk of Phase I trial participation differently from their own personal risk of harm. As we illustrate below, when such risk perceptions diverged, participants typically perceived their individual risk to be lower than the average Phase I trial. More than just the particular clinical trial in which they were enrolled during their interview, participants’ broader trial experiences informed their perceptions. This section presents our findings from healthy volunteers’ perceptions of the overall risks, their personal risk of harm, and demographic patterns in these views of risk.

### Perceptions of the overall risk of Phase I trials

There was wide variation in how healthy volunteers characterized the overall risk of Phase I trials, with some asserting that such studies pose negligible risk of harm and others expressing profound fear about the possibility of death or serious injury. Indeed, as we analyzed participants’ risk perceptions, we found that their beliefs about the overall risk could be grouped on an ordinal scale of low, medium, high, or extremely high risk. This type of quantifying of qualitative risk perception allowed us to account for the prevalence of each view as well as explore demographic differences among groups (see below). Most participants (85 of 178) perceived the overall risk of Phase I trials as medium, followed by those who saw it as high (31 of 178), low (20 of 178), and extremely high (2 of 178) (see **Table 2**). No participants made unqualified assertions about all Phase I trials as being without risk altogether.

**Table 2:** Perception of overall risk.

Risk	*n*	Overall percent	Percent without missing data
**None**	0	0%	0%
**Low**	20	11.2%	14.5%
**Medium**	85	47.8%	61.6%
**High**	31	17.4%	22.5%
**Extremely High**	2	1.1%	1.5%
**Missing**	40	22.5%	–
**Total**	178		*n* = 138

Participants in our low-risk category often emphasized how safe Phase I trials are. They did not believe that very risky trials exist, primarily because they had never heard of or experienced any they considered to be more than low risk. Some participants argued that dangerous clinical trials are not in the interest of the pharmaceutical companies developing the drugs. For example, a white man in his 40s who had participated in 20 studies explained,

Since they [pharmaceutical companies] have millions and millions of dollars riding on them, it’s not very risky because they don’t want to take the risk of not being able to put it on market, and they know that if participants are having bad reactions…, they won’t be able to market it. (F2413)

Additionally, even when participants acknowledged the possibility of adverse effects, they perceived these occurrences as unconcerning problems that resolve quickly.

In comparison, participants in our medium-risk category typically thought the risk depends on the trial in which one enrolls. Rather than seeing Phase I trials overall as moderately risky, they generally envisioned a range of available trials with some being no or low risk and others being high or extremely high risk. In other words, they perceived a diverse spectrum of Phase I trial risks that average out to medium risk. The following articulation from a black man in his 30s who had participated in 20 studies epitomizes the medium-risk position:

You can’t really say [overall how risky it is to participate in Phase I trials]. … You can’t put a gauge on it because it all depends on the study you’re doing and what the side effects are and whatnot. … So, you can’t say it’s risky, you can’t say it’s not risky. And it all depends on the individual and the study, so I can’t really-, I don’t know how to answer that. (F1424)

For these individuals, the uncertain and variable risks of trials ultimately balance out, reaching equilibrium at medium risk.

Unlike participants in the low- and medium-risk groups, participants who perceived the risks as high or extremely high expressed apprehension about the danger of testing investigational drugs. We found, however, that participants in the high-risk group had a more laissez-faire attitude toward those risks than did the participants in the extremely high-risk group. For example, we classified in the high-risk group an African American man in his 20s who had participated in three Phase I trials. He said,

I think it’s very risky. I mean, I just took a chance and maybe I shouldn’t have took a chance because maybe let’s say that, you know, I wasn’t fortunate enough to be here sitting with you here right now. I mean, let’s say I was in the study and then something weird happened, like my body reacted weird to a medicine, and then, you know, I died or like I’m in a wheelchair or something’s wrong with me. But—I don’t know—it’s just your own risk. It’s like, it’s up to you, you know, if you want to do it. (F1318)

In contrast, we categorized in the extremely high-risk group a Hispanic man in his 20s who was in his first study. He believed that being harmed in Phase I trials is almost inevitable:

And it’s a very high risk when they make you sign papers, consent forms, you know. … When you’re signing papers, it’s for a reason. If you’re signing a paper where you can’t sue them if you die or whatever, then that’s real. You know, that’s at your own risk, you know, that’s-that’s-that’s-that’s a red light, you know. So, I do look at this as playing with fire. And you can only play with fire so long before you get burned. … So that’s why I think it’s a huge risk. From a scale of one to a hundred, it’s at a hundred. (F1110)

Participants in the high-risk and extremely high-risk groups viewed the magnitude of Phase I risks to be of great concern without any articulation that some trials are no or low risk. The two groups of participants deviated in that those who thought of trials as high risk discussed their participation as a “choice” they voluntarily made, whereas the extremely high-risk group emphasized the desperate economic circumstances that forced them to accept risks that made them deeply uncomfortable.

### Demographic differences in overall risk perception

The process of categorizing healthy volunteers’ perceptions of overall risk allowed comparison among groups and exploration of how sociodemographic differences influenced their views (**Table 3**). Generally, there were few demographic patterns in participants’ risk perceptions. For example, gender, race, educational attainment, employment status, and household income did not play a statistically significant role in participants’ risk perceptions. We did, however, find that participants who were in their first year of participating in clinical trials were more likely to see clinical trials as higher risk overall than those who had been enrolling in studies longer (Jonckheere-Terpstra = −2.433, *p* = 0.015).

**Table 3:** Demographic differences in perceptions of overall risk.

Demographics	Low	Medium	High and Extreme	Number	Number Total	J-T	*p*-value
**Gender**							
Men	14 (13.3%)	64 (61.0%)	27 (25.7%)	105	138	-	0.603
Women	6 (18.2%)	21 (63.6%)	6 (18.2%)	33			
**Minority Status**							
Non-white or Hispanic	8 (8.8%)	57 (62.6%)	26 (28.6%)	91	138	-	0.016*
Non-Hispanic white	12 (25.5%)	28 (59.6%)	7 (14.9%)	47			
**Ethnicity**							
Hispanic or Latino	0 (0.0%)	18 (62.1%)	11 (37.9%)	29	138	-	0.008*
Non-Hispanic	20 (18.3%)	67 (61.5%)	22 (20.2%)	109			
**Education**							
High School or Less	4 (10.3%)	25 (64.1%)	10 (25.6%)	39	138	−0.691	0.490
More than High School	16 (16.2%)	60 (60.6%)	23 (23.2%)	99			
**Employment**							
No Full-time Employment	18 (15.8%)	68 (59.6%)	28 (24.6%)	114	138	0.221	0.825
Full-time Employment	2 (8.3%)	17 (70.8%)	5 (20.8%)	24			
**Income**							
≤US$25,000	10 (15.4%)	39 (60.0%)	16 (24.6%)	65	137	0.03	0.974
>US$25,000	10 (13.9%)	45 (62.5%)	17 (23.6%)	72			
**Trial Participation**							
First Year	3 (8.3%)	19 (52.8%)	14 (38.9%)	36	138	−2.433	0.015*
Beyond First Year	17 (16.7%)	66 (64.7%)	19 (18.6%)	102			

*Indicates statistically significant results (*p* < 0.05).

Categorical data are presented as number (%); *p*-values were obtained by Fisher exact test.

Ordinal data are presented as number (%); *p*-values were obtained by J-T tests.

Abbreviation: J-T, Jonckheere-Terpstra.

We also found statistically significant differences based on minority status and Hispanic ethnicity (respectively, Fisher exact test, *p* = 0.016; Fisher exact test, *p* = 0.008). Race alone did not generate any statistically significant difference (chi-squared = 1.594, *p* = 0.451), but this appears to be because the racial category of “white” included both Hispanic and non-Hispanic participants. When we analyzed participants in the binary groups of non-Hispanic whites and minorities (i.e., all other racial and ethnic groups combined), we found a statistically significant difference in risk perceptions: minorities viewed clinical trials as higher risk than did non-Hispanic white participants (28.6% versus 14.9%). Additionally, Hispanic participants viewed clinical trials as higher risk overall than non-Hispanics (37.9% versus 20.2%). Thus, the key sociodemographic variables that explain differences in perceptions of overall risks were experience in clinical trials, minority status, and Hispanic ethnicity.

### Perceptions of personal risk of harm

Participants’ perceptions of the overall risks of trials often operated independently from their beliefs about their personal risk of being harmed. While participants acknowledged the possibility they personally could be harmed in a Phase I trial, they had differing views on the likelihood of that occurring. Specifically, we were able to classify 154 (86%) of our 178 participants as believing either (1) that they are safe from harm or (2) that they are vulnerable to being harmed during a clinical trial (**Table 4**). Of these 154 participants, 97 articulated the belief that they would be safe from harm, whereas 57 felt vulnerable to being harmed. We found that participants provided rationales for these beliefs based on their understanding of study risks, level of trust in the research clinics, decisions about studies, and personal or vicarious prior trial experience. We illustrate each of these factors below, according to participants’ perceptions of their personal risk of harm.

**Table 4:** Perception of personal risk of harm.

Perception	*n*	Overall percent	Percent without missing data
**Safe from Harm**	97	54.5%	63.0%
**Vulnerable to Harm**	57	32.0%	37.0%
**Missing Data**	24	13.5%	–
**Total**	178		*n* = 154

### Safe from harm

Participants who felt safe from harm in Phase I trials gave a variety of rationales for this risk perception: they dismissed the seriousness of harms that could occur (“nonchalant”), felt the odds were in their favor (“low odds”), trusted the research clinic would protect them from harm (“trusting”), believed their own good health would keep them safe (“invincible”), asserted they enrolled only in safe studies (“discriminating”), claimed they would withdraw from a study if they had an adverse effect (“willing to quit”), engaged in health behaviors they believed reduced trial risks (“risks are eliminated”), and judged risk based on positive past experiences (“prior evidence”). **Table 5** shows the prevalence of these participant rationales for what kept them safe from harm. These categories were not discrete, and individuals often used multiple rationales to justify their risk perception.

**Table 5:** “Safe from harm” participants.

Rationale for Belief	*n* = 97	Percent
Nonchalant	74	76.3%
Low odds	12	12.4%
Trusting	55	56.7%
Invincible	24	24.7%
Discriminating	55	56.7%
Willing to quit	8	8.2%
Risks are eliminated	23	24.7%
Prior evidence	44	45.4%

Most participants in this group (74 of 97) expressed nonchalance about the seriousness of Phase I trial risks. This manifested not in a denial of the possibility of experiencing adverse effects but in the view that these symptoms were not harmful. For example, when asked about the risks of the trial in which he was currently enrolled, a Hispanic man in his 20s who had participated in six studies replied,

Just side effects, but I mean, they’ll go away once you’re done. … You’ll have side effects if, you know, if you keep taking them [the medication], but once you stop taking them, your body goes back to how it was. (F3428)

Twelve participants thought that the odds of being harmed did not warrant concern. This was voiced by a white man in his 20s who had participated in seven studies:

You know, they’re gonna give you [i.e., they list on the consent form] every effect. You know, they’ll say a side effect is death. [laughs] That doesn’t really deter me ‘cause I don’t think the odds are that’s gonna happen. (P1402)

In addition, many participants who thought they would be safe from harm (55 of 97) justified this belief through their trust in the clinics and/or the larger research enterprise. For example, a white woman in her 30s who had participated in 45 studies claimed,

[For people who have never participated,] Everybody’s like, “Oh, you’re gonna get this [side effect], you’re gonna get-,” you know. But then at the same time, I’m like you’re in the facility with doctors, nurses, and there is really-, you know, nothing can happen to you. And I don’t think they really would, you know, put a really bad drug out there to where, you know, something’s gonna happen to you. (F2410)

In each of these cases, participants perceived that the studies themselves or the research oversight ensured that they would not be harmed.

In addition to evaluating the inherent risks of Phase I trials, many participants thought they were safe from harm in studies because of their own good health or their behaviors. A quarter of these participants (24 of 97) seemed to suggest they were invincible to trial risks. A Hispanic man in his 30s who had participated in 20 studies illustrated this viewpoint:

And in any one of the cases that I’ve done [studies], that I’ve taken myself into, you know, the-the-the mouth of the lion, you could say, I’ve had to trust that my body will take me there [and] that I’m healthy enough to not have complications. Yes, that may be naïve. Yes, I may be full of bravado by saying, you know, “I’m Superman.” But it’s helped me out so far. … I’ve never had complications throughout, you could say, the-the decade or so that I’ve done this. (F1425)

Additionally, more than half (55 of 97) of the participants felt safe from harm based on their reported ability to discriminate between studies and to enroll only in those they deemed safe. This can be seen in how an African American man in his 30s who had participated in 16 studies described his decision-making:

I do the ones that has little to no risk. You know, I’ve actually been explained studies after I call it in to facilities [i.e., research clinics] and they told me the possible risk. Or if I hear the medication, I ask them what it’s for, and if it sounds like something that-that might have a cause and effect on, you know, my body or my mind, then I-, you know, if I feel too iffy about it, so to speak, I wouldn’t participate. (F1439)

Several participants (8 of 97) also claimed they were ultimately safe from harm because they would withdraw from a study should any negative effects occur. For example, a white man in his 50s who had participated in 25 studies declared,

If I start having really bad side effects that are listed, I would not hesitate to quit. *…* Like they say [you could have] dizziness, fainting, vomiting, diarrhea, [so] if I had anything serious start, I would drop [out of the study]. (F2409)

Others thought that their health behaviors, such as their diet or exercise, also contributed to making it through a clinical trial unscathed. Specifically, 23 of 97 participants believed they could eliminate the risk of harm by flushing the investigational drugs out of their bodies, eating a healthy diet, or consuming vitamins or supplements. For instance, a Hispanic man in his 30s who had participated in 10 studies explained why he was not concerned about being harmed,

Just because like I know when I go home, I’m going to live this healthy lifestyle. You know, just eating really good, lots of vegetables, greens, and I do kind of like these herbal remedies, like, to detox and to lower my cholesterol and everything. (F3461)

In each of these instances, participants assumed that they were personally shielded from harm (even if others could be harmed in the same trials) or that they could mitigate harm through the choices they made about studies or by managing their health.

A final factor in why 45% of the participants (44 of 97) believed they were safe from harm in studies was based on their own or others’ prior experiences in Phase I trials. Illustrating this view, a Hispanic man in his 40s who had participated in six studies said,

I haven’t been concerned [about the risk] to this day because I’m aware of what the effects of the medications are when one participates in a clinical study. And I know some of those reactions can be bad for your health, you know, but I have not experienced anything severe, nothing serious to this day, so I have no concerns about it. (F3427, translation from Spanish)

Additionally, because most Phase I trials require a confinement period, participants had met other healthy volunteers who had enrolled in many more studies over a much longer period of time [40]. The health of these study “veterans” also served as proof that participants would be safe from harm. A Hispanic woman in her 40s who had participated in three studies professed,

I ended up finding, you know, a guy. He’s been here [participating in studies] like for 15 years. I said, “Oh, you still look normal.”… So, and everybody that I see here [at the research clinic], I mean, I don’t see, you know, anything wrong [with them]. They keep coming back, so obviously for one reason or another, I mean, it looks like they’re maintaining healthy [sic] and the medicine’s not that big of a deal. I don’t know. That’s now my aspect of seeing things. (F3315)

In these instances, participants used this prior experience as evidence that they would not be harmed by enrolling in Phase I trials.

### Vulnerable to harm

For the 57 out of 178 participants who believed they were personally vulnerable to harm from their Phase I trial participation, there was variation in how they assessed their vulnerability. Some expressed great concern about being injured while others were quite uncertain what might happen to them. As rationales for their beliefs, they typically focused on the possibility that they could experience distressing adverse effects (“susceptible”), their reaction to the study drug was impossible to predict (“incalculable”), the clinics might not be fully trustworthy (“distrusting”), and/or their prior experience with adverse effects indicated that problems in studies routinely occur (“personal experience”). These were not mutually exclusive categories, and individuals provided multiple rationales to explain their vulnerability to harm (**Table 6**).

**Table 6:** “Vulnerable to harm” participants.

Rationale for Belief	*n* = 57	Percent
Susceptible	34	59.6%
Incalculable	11	19.3%
Distrusting	18	31.6%
Personal experience	20	35.1%

Nearly 60% of participants who felt vulnerable to harm (34 of 57) expressed concern about their personal susceptibility to clinical trial risks, either articulating the conviction that any study poses the threat of harm or that any experience of side effects is unwanted and concerning. An example of the former came from a Hispanic man in his 30s:

Every time you do it [enroll in a clinical trial], you’re taking some risk. … Like, I’ve come in here like seven times, but I mean, each time I’d do it, I was like, you know, “I know I’m taking like a risk coming in here,” you know. Like, even the doctors tell you… there’s a chance if you’re allergic to it, you could die, you know. … It’s definitely not for somebody if you’re like really obsessed with your own mortality, you know. And I don’t see how you cannot be. (F3459)

This participant’s anxiety about the possibility of dying was palpable in the interview, but even when other participants were not focused on the risk of death, they expressed concern about adverse effects. An African American man in his 20s who had participated in four studies proclaimed,

I really don’t want none [side effects], to be honest with you, but maybe a slight headache or something like that. But that’s it. I don’t want no nausea, you know, nothing that’s going to make me vomit, diarrhea, nothing, nothing crazy like that. (P1301)

This view is in contrast to the participants who thought of themselves as safe from harm and were nonchalant about these very same side effects of vomiting or diarrhea. In other words, the view of personal harm is not necessarily anchored to specific symptoms but could instead be based on whether participants perceive those symptoms as signs of harm.

A smaller portion of participants in this subsample (11 of 57) found it difficult to predict what might happen to them, and this uncertainty was the basis for their sense of vulnerability to harm. For example, a Hispanic man in his 40s in his first study explained,

They’re using you to, you know, find out any adversity towards the medication. … They’re using humans, you know, instead of using the mouse, mice… And so, you know, it carries some type of risk ‘cause… you don’t really know what type of side effect that you’re going to get until you actually do it. ‘Cause, you know, you’re not… a future reader, you know, so you can’t foretell the future until it happens. (F1126)

Other participants stated that trial consent forms detail what could happen, but there is no way to know what will actually happen. This led to some anxiety as they waited to see whether any symptoms would manifest during a trial.

A notable minority of participants who felt vulnerable to being harmed in a clinical trial (18 of 57) expressed distrust in Phase I research clinics or in the larger system of research oversight. A Hispanic man in his 30s enrolled in his first trial confided,

There’s rumors going around that other people are sick in here right now… Staff’s been saying nothing been in here deadly, [that] nobody’s gotten too sick, you know. And I feel like some people will be telling the truth, and they could be telling the truth because… they’re telling me only what *they* know. And then some people could be just telling you because they need obviously—you know, for them to get paid—they need people coming here to get studies. So, a person would lie to you in a heartbeat. Can’t trust everybody. (F1110)

Participants like this had concerns that consent forms contained only partial information or that the clinic’s financial bottom line took priority over participants’ safety.

Many of the participants concerned about being harmed in studies (20 of 57) based this worry on their prior experiences or on adverse effects they had witnessed or heard about happening to other healthy volunteers. As one such example, an African American woman in her 30s enrolled in her first trial commented,

When I came into the study,… I was told like about 50 to 60% of people actually see the side effects. So I-I guess I didn’t go into it thinking, “Okay, I’ll be the percentage that *doesn’t* get side effects.” I assumed the worst, that I *would* get the side effects, so it didn’t really surprise me [that I did]… I think I would look at it the same way for the next study. (F2109)

In other words, the experience of having unpleasant, even if only short-term, adverse effects made some participants feel more vulnerable to this happening in any Phase I trial.

### Beliefs about personal harm relative to perceptions of overall risk

As might be expected, participants’ perceptions of the overall risks of trial participation and their perceptions of their personal risk of harm were correlated (chi-squared = 34.041, *p* < 0.0001) (**Table 7**). Specifically, all participants who thought trials were low risk believed they were safe from harm, and all those who perceived trials to be extremely high risk felt vulnerable to harm. The remaining participants who viewed trials as being medium or high risk did not have a clear pattern distinguishing them from each other in their belief about personal harm. Some believed they were safe from harm while others felt vulnerable to harm. Those who thought they were safe from harm were particularly likely to use rationales appealing to their own health or health behaviors to explain why they would not be harmed while others participating in Phase I trials might be. Indeed, 74% of the participants who felt safe from harm despite perceiving trials as medium or high risk overall (46 of 62) believed that they could identify and enroll exclusively in low-risk studies. Interestingly, even for those participants who felt vulnerable to harm, 72% (41 of 57) had narratives that described similar techniques of avoiding risky studies and engaging in health behaviors to reduce their risk.

**Table 7:** Distribution of perceptions of personal risk of harm by perceptions of overall risk.

Risk Perceptions	Safe from Harm *n* = 97 (% of group)	Vulnerable to Harm *n* = 57 (% of group)	Missing Personal Risk Data
**Low**	20 (20.6%)	0 (0.0%)	0
**Medium**	54 (55.7%)	22 (38.6%)	9
**High**	8 (8.2%)	22 (38.6%)	1
Extremely High*	0 (0.0%)	2 (3.5%)	0
**Missing Overall Risk Data**	15 (15.5%)	11 (19.3%)	14

*Because of small sample size, chi-squared testing was done by combining high and extremely high risk.

### Demographic differences in beliefs about personal harm

There were no statistically significant demographic differences in how participants perceived their personal risk of harm (**Table 8**).

**Table 8:** Demographic differences in perceptions of personal risk of harm.

Demographics	Vulnerable to Harm	Safe from Harm	Number	χ^2^	*p*-value
**Gender**					
Men	43 (38.7%)	68 (61.3%)	111	0.508	0.476
Women	14 (32.6%)	29 (67.4%)	43		
**Minority Status**					
Non-white or Hispanic	40 (39.6)	61 (60.4)	101	0.845	0.358
Non-Hispanic white	17 (32.1)	36 (67.9)	53		
**Ethnicity**					
Hispanic or Latino	17 (45.9%)	20 (54.1%)	37	1.667	0.197
Non-Hispanic	40 (34.2%)	77 (65.8%)	117		
**Education**					
High School or Less	18 (42.9%)	24 (57.1%)	42	0.846	0.358
More than High School	39 (34.8%)	73 (65.2%)	112		
**Employment**					
No Full-time Employment	46 (36.8%)	79 (63.2%)	125	0.013	0.910
Full-time Employment	11 (37.9%)	18 (62.1%)	29		
**Income**					
≤US$25,000	31 (44.3%)	39 (55.7%)	70	3.284	0.070
>US$25,000	25 (30.1%)	58 (69.9%)	83		
**Trial Participation**					
First Year	21 (48.8%)	22 (51.2%)	43	3.578	0.059
Beyond First Year	36 (32.4%)	75 (67.6%)	111		

## Discussion

Similar to prior research on risk perceptions [26], our findings demonstrate that risk perception is multifaceted, with healthy volunteers perceiving the overall risks of clinical trials and the risks of personal harm as generally different. The majority of our participants characterized Phase I trials to be medium or high risk, but most nonetheless assumed they were personally safe from harm. Importantly, the dominant belief among participants that they are unlikely to be harmed is broadly reflective of the meta-analyses that support the relative safety of Phase I trials in terms of the risk of serious or life-threatening problems, although not in avoidance of unpleasant side effects [1–3].

Going beyond prior qualitative research on risk [27,28], our quantification process revealed that racial and ethnic minorities, particularly Hispanics, tended to view the overall risks of clinical trial participation as higher, but neither minority status nor ethnicity yielded statistically significant differences for personal risk of harm. In other words, even though these groups largely perceived Phase I trials as riskier than did other participants, they did not appear to feel personally more at risk of being harmed than any other group. One interpretation of this tendency is that minorities are more likely to have concerns about research based on past abuses of African Americans, Hispanics, and other disadvantaged groups [41–43], but firsthand experience in Phase I trials diminishes their personal view of risk.

Our quantitative analysis also found that healthy volunteers who were new to Phase I trials perceived the overall risks as higher than did individuals who had participated in studies for more than a year. However, as with our findings for race and ethnicity, there were no statistically significant differences in perceptions of personal risk of harm based on participants’ clinical trial experience. Surprisingly, no other demographic characteristics—such as gender, educational attainment, employment status, or household income—seemed to affect perceptions either of overall risk or personal risk of harm. This underscores the importance of prior experience in Phase I studies on healthy volunteers’ risk perceptions. It could be inferred that participants new to clinical trials hold risk perceptions that are based on a limited understanding of the types of risks to which participants might be exposed. First-time participants’ views of Phase I trials may even be informed by fictional portrayals of medical research as a highly risky activity [44,45]. Once enrolled, they experience the various ways that risks to participants are minimized or mitigated in clinical research.

Most importantly, our qualitative findings provide an explanation for why participants might perceive the overall risks of trials as distinct from their own personal risk. Specifically, participants viewed studies as having differential risks, interpreted the experience of symptoms disparately, had divergent levels of trust in the clinics and/or research enterprise, understood individuals’ susceptibility to harm as variable, and attempted to assert differing levels of control over the risks. Of course, participants’ risk narratives were not all equally accurate in tracking their personal risk of harm. Some participants’ rationales for their views of why they were personally safe from harm might have been based on false assumptions that the choices they made actually diminished their risk, that their own good health truly could protect them, or that withdrawing from a study could reverse any adverse effects that had commenced. Likewise, for participants who felt vulnerable to harm, their worries about the impossibility of anticipating how participants would fare in trials and the involvement of nefarious researchers who cared more about money than protecting participants were similarly problematic accounts that exacerbated participants’ sense of personal risk.

At the aggregate level, however, the complexity of these narratives illustrates how participants’ perceptions of risk—at the overall trial or personal level—were multifactorial and constituted through individuals’ experiences in studies, the choices they made about enrollment, and their degree of comfort with participating in clinical trials. It could be argued that the majority of participants did not believe they would personally be harmed because they were invested in promoting a sense of control over risk-taking associated with their trial enrollment. Particularly for serial participants, assertions of such control may have been a significant factor in their continued Phase I involvement, whether or not such a sense of control was warranted.

The variable and complex factors that contributed to participants’ risk perceptions elucidate why there are no simple solutions to the ethical problems these perceptions raise. For example, it may seem ethically inappropriate to include participants who view clinical trials as holding extremely high risk and yet enroll because of economic desperation. If the compensation distorts such participants’ risk perceptions, a concern about undue inducement is ethically significant [25,46]. However, it is also the case both that risks are not necessarily in fact higher for these participants and that any effort to exclude such participants from trials may fail in ways that are also ethically problematic. Healthy volunteers, after all, are savvy about qualifying for clinical trials even, or perhaps especially, when they enroll out of dire financial need [47].

Our study has several limitations. First, we are presenting quantified versions of risk perceptions based on a method of categorizing and aggregating data from in-depth interviews. This means that our overall risk scale and assessments of participants’ views of their personal risk of harm are based on our interpretations of participants’ narratives regarding risk and decision-making. Thus, we do not know whether the participants themselves would agree with how we categorized them. We also have missing data for participants who simply could not be categorized based on the information in their interview transcripts. The missing cases were primarily due to participants’ terse responses to interview questions, but in several cases particular questions about risk in the interview guide were not asked. A second limitation comes from our sampling technique. Although we enrolled participants who were demographically diverse from seven Phase I clinics, we did not have a random sample, which makes our results less generalizable. Finally, our sample size—while robust for qualitative research—was limited for more complex quantitative analyses. Despite these limitations, the overall strength of our qualitative approach is that it allowed for the emergence of participant-centered risk perceptions, as well as discussions about how their own actions might mitigate those risks. This would have been difficult or perhaps impossible to achieve with a survey-based approach. In quantifying in-depth interviews, we minimized some of the potential biases of survey responses, including tendencies toward extreme answers (extreme responding) or question-order bias. Yet, social desirability bias remains a possibility with interview data.

## Conclusion

Our study demonstrates that healthy volunteers are generally reflective about the risks of Phase I participation and distinguish between the overall risk of Phase I trials and their personal risk of harm. Racial and ethnic minorities as well as participants new to clinical trials typically saw the overall risks of trials as higher than did non-Hispanic whites and participants with more trial experience, respectively. However, there were no differences among groups in their perceptions of personal risk of harm. The discrepancy in healthy volunteers’ views of overall and personal risk sheds light on why healthy volunteers might continue to enroll in clinical trials even when they view trials on the whole as risky. While our goal was not to evaluate the accuracy of either set of perceptions, it cannot be the case both that clinical trials overall present significant risk of harm and yet each individual participant is safe from harm. More work needs to be done—whether for Phase I trials on healthy volunteers or later-phase trials on affected patients—to determine how participants close the gap between their perceptions of overall clinical trial risks and personal risk of harm. This is important in part because relying on improbable narratives of personal exemption from risk of harm may indicate that participants are unduly induced by the offer of compensation and that the voluntariness of their participation is therefore compromised. In future empirical studies, if researchers conflate the potentially differing views of risk, they may also fail to illuminate problems in how participants construct narratives of clinical trial risk or personal risk exemption, or in how clinical investigators present or diffuse risk information.

## Supporting information

S1 Checklist Consolidated Criteria for Reporting Qualitative Research (COREQ). (DOCX)

S1 Appendix Semi-structured interview guide. (DOC)

## Abbreviations

COREQ: Consolidated Criteria for Reporting Qualitative Research
PI: principal investigator
