# Supplementary material for: Healthy volunteers' perceptions of risk in US Phase I clinical trials: A mixed-methods study
Source: PLoS Med. 2018 Nov 20;15(11):e1002698. doi: 10.1371/journal.pmed.1002698 (PMC6245523; doi:10.1371/journal.pmed.1002698)
Supplement: S1 Appendix — (DOC) [file pmed.1002698.s002.doc]

**Semi-Structured Baseline Interview Guide**

*Note: The interview guide represents a full set of potential interview questions, which might not apply to all participants, such as first-time healthy volunteers. The interviewer will adapt the guide to best fit the experience-level of the participant and ask prompts as appropriate. The interviewer should have the respondent’s demographic data sheet in hand before starting the interview.*

1. **Background information about healthy volunteer**

*To start off, we are going to go through this questionnaire to get a better understanding of your personal situation.*

- You noted that you live in (street and/or city). How long have you lived at this address? In this city? Where else have you lived prior to your current location? Where were you born?
- You said you are (civil status) and have (# of children). How long have you been in your current civil status? How old are your children?
- How many people live in your current residence including yourself? Who? Do you live with your family members? By yourself? With other people? If have children and not in household, where do your children live? If children reside elsewhere, how often do you send money or gifts to them? How often do you see your children?
- You said you have (level of education). Tell me more about your education. Why did you leave school? What did you study?
- I see that you are (employment status). How long? What kind of work do you do? What was your last job?
  - You said that you lived with (your partner). Is your partner employed? What kind of work does he/she do? What share of your household income is he/she contributing?
  - How does income from clinical trial participation fit into your overall income? (personal and household)
  - How would you characterize your financial stability over the past year? Over the last few years? Changes? Challenges? Stressors?

1. **Information on healthy volunteer’s current study**
   - Tell me about the study that you are taking part in now at this clinic.
     - What type of study is it? How long is the study? How long have you been participating in this study? Tell me about the study schedule and protocol.
     - How did you find out about this particular study? What did you have to do to qualify for the study? [Did you have concerns about qualifying?] Once you heard that you had qualified, what made you decide to participate?
     - What would you say is your main reason for participating in this study? What are the benefits for you?
     - What do you consider the risks of participating in this study? In this study, what are the possible side effects? How concerned are you about these risks? [Have you experienced any side effects as part of the study?]
2. **Background information on healthy volunteer’s experience participating in Phase I trials**
   - How did you ***first find out*** about participating in medical research? How long ago was that?
     - How long did you consider it before enrolling in a study? What made you decide to do it?
     - (If told by someone they know) What did that person tell you that made you want to participate? (If a friend) How long have you known this person? What questions did you ask them about participating?
   - What ***worries or concerns*** did you have initially about being in a study? How have those concerns changed since that first study? What would you say has made them change?
   - *(If participated in 2-5 studies)* Tell me about the other studies you have done. What types of studies were they? Where?
   - Tell me about some ***memorable studies*** that you have done. What made them memorable?
     - What procedures or elements of studies have surprised you?
     - When thinking about past studies, what was the most difficult aspect of participation in a previous study?
   - What do you consider the ***risks*** of participating in studies like these?
     - Based on all of your experiences participating in clinical trials to date, how risky do you think it is to enroll in studies like these? Why?
     - Would you characterize the risks as short-term or long-term? Why? (If both,) how do the risks differ in the short or long terms?
     - How have your perceptions of the risks of studies changed based on your experiences?
     - Other than bodily risks, what are other risks to you of being in studies? (To your personal or social life? To your employment?)
   - What do you consider the ***benefits*** to you of participating in clinical trials?
     - If benefits are financial, to what uses do you put the money you earn in studies?
     - How have your perceptions of the benefits changed based on your experiences?
     - Other than money, what would you say you get out of doing studies? (Benefits for your health? For your personal or social life? For society?) How important are these other benefits to you?
   - Tell me about the ***clinics*** in which you have participated in studies. How do they compare to each other?
     - Are there clinics you would prefer to do studies at? Why? What about clinics you prefer not to go to for studies? Why?
     - Tell me about being confined to the research facility. How does that affect you? How does it affect your family?

- What has been your experience of the study regimens? Tell me what it’s like to be told what to do and where to be. What is the hardest part about this?
  - - Generally, ***how safe*** do you feel in these clinics? How confident are you that you are being well cared for? What gives you these impressions?
      - Tell me about a time in which you ***felt your safety was compromised***. What was going on? How did you respond? Do you feel that you could bring up your concerns to staff/doctors or other participants if you felt unsafe?
  - Tell me about your ***interactions with research staff***.
    - How does this differ by clinic? (Physicians / nurses / coordinators / recruiters / other staff?)
    - Tell me about a particularly good experience you had with study staff. Have you ever felt as though a staff member is your advocate?
    - Tell me about a particularly negative experience you had with study staff. Did you ever consider not enrolling or dropping out of a study because of an issue with the staff’s ethics / professionalism? Who do you talk to when you have negative experiences in studies? What do you tell them?
- Have you seen or heard about instances where staff treat volunteers differently? According to race/ethnicity/gender/age? Where? What did you hear? (based on gender, race, ethnicity, age, clinical trial experience level, etc.).
  - Tell me about ***your interactions with other participants*** during studies.
    - Who do you tend to get along with?
    - What kinds of relationships or friendships have you made by participating in studies? How important are these relationships to you? To what extent do these relationships continue when a study ends?
    - How much do you share information about studies with other participants? How do other participants influence your decision to participate in specific studies?
    - Who don’t you get along with? Tell me about a negative experience you have had with another participant during a study. What happened? Who do you talk to when you have negative experiences with other participants in studies? What do you tell them?
    - Tell me about the group dynamics. What groups form during studies? (by race/ethnicity, language, gender, age)
    - Which types of people tend to be easy-going in studies? Which types of people tend to create trouble or conflict in studies?

1. **Decisions about trial participation**
   - ***How often do you screen for*** studies? Tell me about how you look for a new study to participate in. What process do you go through to find one?
     - *1^st^ time participants*: I know this is the first study you enrolled in, but before this study, had you screened for previous studies? What happened that you didn’t participate in them?
   - How ***easy is it to qualify*** for studies? Have you ever failed a screening? What happened?
     - Have you ever ***declined to participate*** in one that you heard about when you called a clinic? What about one that you screened for? Tell me about why you decided not to do that study (those studies).
     - How often do you think people decide not to do studies after screening for them? Why do you think people might decide not to participate in studies?
   - How would you ***decide whether or not*** to do a study?
     - What types of studies do you prefer to participate in? What types of studies are you unwilling to participate in?
     - Which studies do you perceive to be riskier than others? Why? What factors help you to know that a study is riskier than others?
     - What are the risks or side effects that you would be willing to tolerate? What are the risks or side effects that would make you decide not to do a study? Why?
     - Is there one particular risk that makes you particularly nervous (when considering study participation)?
     - What types of medical procedures are you unwilling to undergo? Why?
     - How willing are you to provide a genetic sample as part of a study? Why?
     - What types of drugs would you hesitate taking in a study? How would you decide whether or not to do the study?
     - How does the length of the study influence your willingness to participate? Why?
     - What would be the longest study you would participate in? Why?
     - What other factors could make you decide not to do a study?
     - How far are you willing to travel to do a study? How do you decide if you will screen for a study that is outside your hometown?
     - What has influenced your thinking about what you are willing to do and not do in studies?

- How could the ***amount of compensation*** make you change your mind about these preferences? Which preferences do you have that no amount of money would make you change your mind?
  - What other factors do you think would make you change your mind about these preferences?
  - What is the most money you have made from participating in a single clinical study? (Tell me more about that study. Why do you think it paid more than other studies you’ve done?) What is the least amount of money you have made from a study?
  - How fair is the compensation you receive for doing studies? Why?
  - How difficult is it to participate in studies given your other ***work obligations***?
    - How much about your participation in studies does your boss know? What about your co-workers?
- What about ***family obligations***?
  - - Who takes care of your kids (if any), pets, etc when you are away? How does that affect your relationship with that person?
    - How do you explain to your friends and family why you are away for certain periods of time?
    - During a study, how often do you communicate with friends or family? Who? What do you discuss?
    - How does the separation from your family affect your relationship with them? (Is there something that you have missed due to study participation? [birthday, holiday, graduation, etc.]) How have you felt in these situations? How have you coped with these situations?

1. **Behaviors related to trial participation**
   - *Now, we are going to ask you about some of your behaviors between studies, like diet, exercise, and alcohol consumption. Some of these questions are sensitive, so I’d like to remind you that everything you tell us is confidential and will not be shared with the research staff here or any other group.*
   - Since participating in these studies, **how has your health changed**? Has it improved, stayed the same, or worsened? How?
   - What are the ***things you do between studies*** so that you will be able to qualify for the next study?
     - How much exercise do you tend to get in an average week? Has your amount or type of physical exercise changed since you started participating in studies? How?
     - How would you describe your current diet or eating habits? How has your diet changed since you started participating in studies?
     - How often do you take vitamins or supplements? What kinds do you tend to take? How has your consumption of vitamins or supplements changed since you started participating in studies?
     - How much alcohol do you tend drink in an average week? How has your consumption of alcohol changed since you started participating in studies?
     - How often do you smoke cigarettes? How has your participation in studies affected how much you smoke?
     - How often do tend to use prescription drugs? How often do tend to use recreational drugs? How has your use of prescription and recreational drugs changed since you started participating in studies?
     - Have you been sexually active since participating in studies? Has your method of contraception or birth control changed since you began participating in studies?
     - Are there things you do to ***minimize the effect of the study drug during studies***? **(after studies?)** Why? Have you heard about things that other healthy volunteers do? How effective do you think those things are?
     - How seriously do you take the ***washout period*** for studies? Why? Are there circumstances that would make you decide not to observe it?
     - How seriously do you take other restrictions on participation? (Number of procedures? Types of drugs?) Why? Are there circumstances that would make you decide not to observe them?
   - Have you ever had to stretch the truth or withhold information to get into a study? Tell me about some instances in which you had to stretch the truth or withhold information from staff so that you could get into a study. How common is this for you? What information are you most likely to withhold? What information do you always give fully and truthfully? Why?
2. **Framing participation**

- Who do you tend to talk to about your study participation? What kinds of things do you talk about?
  - What do your [other] friends and family think about you participating in these types of studies? (If you haven’t told them) What do you think they would say if they knew?
- Have any of your friends or family members participated after you told them about studies? What information did you give them? How did you explain what you are doing to them?
- How would you explain to someone you know what pharmaceutical companies are trying to learn from the studies that you are in? Why do they need to take so much of your blood? What data do they get from other procedures? What is the reason you are supposed to “washout” between studies?
- Some participants have the identity of a “professional lab rat” or “professional guinea pig,” what do you think makes someone a professional study participant? How do you think of your participation in studies? What makes you different from these other participants?
- **How long do you plan on participating in clinical trials**? Why?
  - How likely are you to keep participating in studies for the next 1 year? 5 years? 10 years? 15 years?
  - What would be the circumstances that would get you to stop participating?
  - Do you currently have an “exit strategy” for stopping your participation in studies? What is it? How likely are you to stick to it?

Structured Probing questions (For use throughout the interview)

In general, if participant has no individual experience for a question, expand to whether they have 1.) witnessed that experience firsthand, or 2.) have heard from others about that kind of experience.

*If lacking experience…*

- Have you heard about….

*With particularly good or bad experiences with research staff or other participants*

- Have you witnessed…other participants having a bad experience with research staff/other participants? What happened? How was the issue resolved?
- Have you heard about… other participants having a bad experience with research staff/other participants? What did you hear?

*After deviant or stressful experiences…*

- How did you feel during that experience? How did you deal with those feelings?
- Who did you talk to about…?  What did you tell them?

General probes
What was going on? How did you respond? How did other people respond?

What exactly did you mean by….?

Could you give me an example of when you did.…?

Tell me about a time when you….

And how did you feel about that….?

Do you remember what you were thinking when....?

How would you go about.....?  How would someone else go about...?

Could you tell me what happened when….?
